# Long-Term Enrollment in Cardiac Rehabilitation Benefits of Cardiorespiratory Fitness and Skeletal Muscle Strength in Females with Cardiovascular Disease

**DOI:** 10.1089/whr.2021.0055

**Published:** 2021-11-29

**Authors:** Mike Pryzbek, Maureen MacDonald, Paul Stratford, Julie Richardson, Angelica McQuarrie, Robert McKelvie, Ada Tang

**Affiliations:** ^1^ School of Rehabilitation Science and McMaster University, Hamilton, Ontario, Canada.; ^2^ Department of Kinesiology, McMaster University, Hamilton, Ontario, Canada.; ^3^ McMaster Physical Activity Centre of Excellence, Hamilton, Ontario, Canada.; ^4^ Department of Cardiology, Western University and St. Joseph's Healthcare Centre London, London, Ontario, Canada.

**Keywords:** long-term cardiac rehabilitation, fitness in women, exercise training

## Abstract

***Background:*** The benefits of short-term cardiac rehabilitation (CR) for improving fitness are well known, but the effects of long-term maintenance-phase CR are less well established. Moreover, changes in cardiorespiratory fitness (CRF) and muscle strength with long-term CR have never been examined specifically in females, a population that is under-researched and under-represented in cardiovascular research. The objective of this retrospective pilot study was to estimate changes in CRF and muscle strength in females enrolled in a long-term CR program.

***Methods:*** Data from 39 females (mean ± standard deviation age 65 ± 9 years) enrolled for at least 1 year in a maintenance-phase CR program were analyzed. The program consisted of aerobic and resistance training, and data were collected annually for CRF (peak oxygen consumption [VO_2_peak, mL/kg/min]) and skeletal muscle strength (one-repetition maximum tests for chest press, seated row, and knee extension, kg). Mixed-model analyses were used to determine changes in CRF over the 5-year follow-up (203 observations) and muscle strength over 6 years (108 observations).

***Results:*** The CRF increased in females by 1.8%/year over 5 years of CR enrollment, and muscle strength increased by 0.6%–2.1%/year over 6 years. These findings are in contrast to the expected age-related declines in fitness over time.

***Conclusion:*** The positive long-term benefits on CRF and muscle strength in females provide initial preliminary support for maintenance-based CR, especially given that this population is commonly under-researched and under-represented in the CR literature.

## Introduction

It has been long understood that the global prevalence and mortality rates of cardiovascular disease (CVD) were higher in males than females, but more recent data suggest that the gap between the sexes is narrowing.^1^ Despite similarities in disease prevalence, however, there remain differences in courses of care and health outcomes between women and men. Women are less likely to be investigated and treated for CVD^2–4^; they are diagnosed 10–20 years later,^2^ present with more severe disease,^3^ and have poorer prognoses^3–6^ compared with men.

Exercise-focused cardiac rehabilitation (CR) is well established as a core component of the continuum of care for individuals living with CVD. Evidence from a large systematic review of 63 studies and 14,486 participants provides strong support for short-term CR programs, typically lasting <12 months, for reducing cardiovascular mortality and hospital admissions in both men and women.^7^ Importantly, these reductions in cardiovascular morbidity and mortality are associated with analogous improvements in cardiorespiratory fitness (CRF)^8–11^ and skeletal muscle strength.^11–15^

Despite the strong evidence supporting CR, females have only represented 15% of data, and study results are usually not disaggregated by sex to allow sex-specific effect sizes to be determined.^7,14,16^ Promisingly, of the few studies that did disaggregate by sex, improvements in CRF after short-term CR programs were reported to be equal,^17,18^ if not greater,^19–22^ in females compared with males. With growing awareness and recognition of the importance of sex and gender considerations in health research,^23^ a meta-analysis was recently attempted to examine sex-based differences in the effects of CR on mortality and morbidity but ultimately was not undertaken because only 2 of the 80 studies provided data for females.^24^

For long-term CR programs, a small body of evidence has reported improvements in fitness,^25–27^ providing support for these programs as important opportunities for not only maintaining fitness but also reducing morbidity and mortality in individuals with CVD. To date, however, no studies of long-term CR have exclusively examined changes in fitness among females. Thus, the objective of this pilot study was to describe long-term changes in CRF and skeletal muscle strength in females enrolled in at least 12 months of a maintenance-phase CR program.

## Materials and Methods

This study was a retrospective chart review of data for members in a community-based, maintenance-phase, exercise-focused CR program. The study was approved by the Hamilton Integrated Research Ethics Board (#2017-1248) and utilized available data from program inception (January 1985) to December 2016. Participants provided consent for their data to be used on initiation into the program.

Data were extracted from January till April 2017. Demographic information of age (years), height (cm), weight (kg), date of cardiovascular event or surgical procedure, time since event (time since cardiac event o program enrollment, in years), reason of enrollment, date of exercise test(s), and duration of enrollment (years) were extracted.

### Study eligibility

For this analysis, we included participant data if they were female, enrolled for at least 12 months in the CR program, and had CRF data from at least two cardiopulmonary exercise tests conducted using cycle ergometry.

### CR program

The CR program was a maintenance-phase, exercise-based program offered to community-dwelling adults ≥18 years old with CVD. Participants were referred to the program by their internal medicine specialist or family physician.

Aerobic training began immediately on program entry, but initiation of upper body resistance training was delayed 6–8 weeks for those individuals recovering from cardiac surgery per published guidelines.^28^ Exercise was individually prescribed. Aerobic exercise was prescribed at least 2 days/week for 30 minutes at 60%–65% of heart rate reserve. Resistance training loads were prescribed at 30%–40% and 50%–60% of 1-repetition maximum (1RM) for upper and lower extremity major muscle groups, respectively, and performed for 1–3 sets of 12 repetitions.

Blood pressure, heart rate, and rate of perceived exertion were recorded at the beginning and end of the exercise sessions for all participants, and they were monitored more frequently as needed if symptoms were observed. Exercise intensity and duration were progressed throughout enrollment per published guidelines.^28^ Members were encouraged to attend the program at least twice weekly, and to add aerobic exercise outside the program to meet physical activity guidelines of 150 minutes of moderate-vigorous activity per week.

Attendance to the program was not regularly monitored. Adherence records were not available. Self-management and heart-healthy living education was provided informally via brochures and knowledge translation seminars, in which program staff would provide ongoing advice and information for maintaining cardiovascular health and answer member questions.

### Study outcomes

The CRF was the primary outcome of interest. The CRF was measured by using cardiopulmonary exercise tests conducted annually and represented by peak oxygen consumption (VO_2_peak). Each datapoint for VO_2_peak was considered one observation. Exercise tests were conducted by using cycle ergometry, and VO_2_peak (mL/kg/min) was measured by using breath-by-breath indirect calorimetry averaged over 15 seconds (Vmax CareFusion, Vyaire Medical, Mettawa, IL).

All tests were conducted by a cardiovascular technologist with physician supervision. Medications were not withheld. Workload started at 100 kpm/min and increased by 100 kpm/min every minute until test termination. Heart rate (beats/minute) and blood pressure (mmHg) were also measured at rest, at peak, and throughout the test.

Upper and lower body skeletal muscle strength outcomes were available for a subset of members (*n* = 19/39, 49%) who attended annual assessments at a testing site that possessed the necessary equipment (Hydrafitness Omnikinetics, Belton, TX) to conduct the tests. Muscle strength was quantified by using 1RM (kg) for chest press, seated back row, and knee extension. Standardized procedures to determine 1RM were followed.^28^ Each datapoint for 1RM was considered one observation.

Data for VO_2_peak, 1RM for chest press, seated back row, and knee extension, and resting and peak heart rate and blood pressure were extracted from program members' charts for all available time points.

### Statistical analysis

All analyses were performed by using Stata 14 (StataCorp, College Station, TX). Descriptive statistics (means ± standard deviation) and frequencies (*n*, %) were used to describe demographic information and baseline characteristics.

Mixed-model analyses for longitudinal data were applied to examine the relationship between changes in the dependent variables (VO_2_peak and 1RM chest press, seated back row, and knee extension) and the independent variable (enrollment time, years), controlling for age at the time of enrollment.

Models were explored for linear and non-linear growth based on the findings of the Lowess curves and likelihood ratio tests indicating the best model. Tests of interactions between enrollment time and age were performed. Relevant random effects and the most appropriate covariance structure were identified. The Bayesian Information Criteria were used to identify the best fitting model.

## Results

**FIG. 1.**
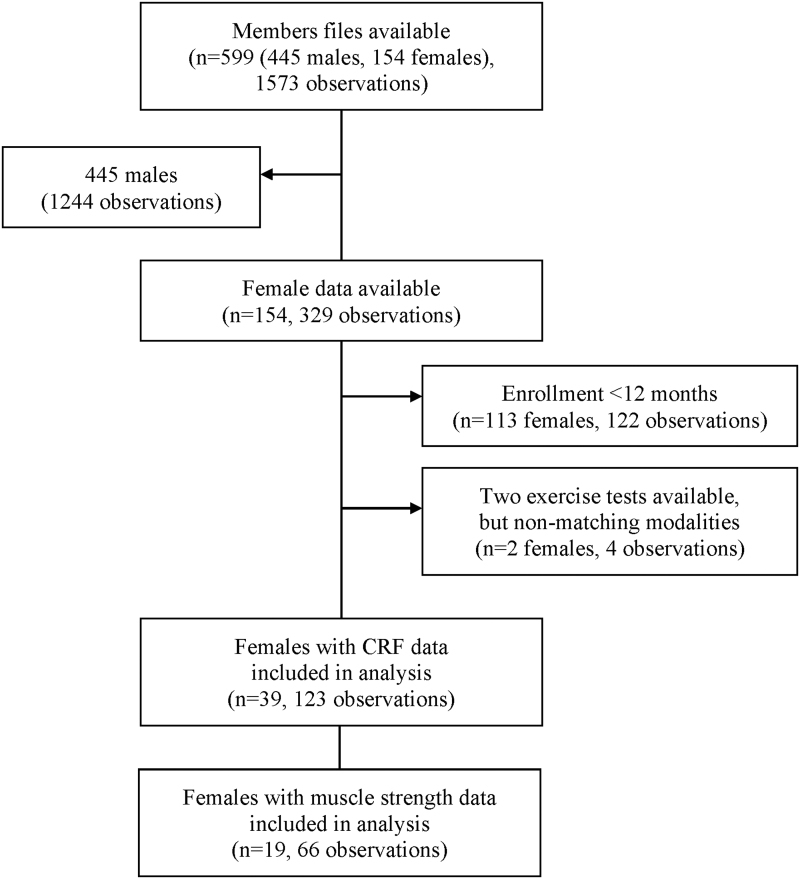
Flow of data extracted and included in the analysis. One observation is a single measurement of cardiorespiratory fitness (VO2peak) or muscle strength at any point in time.

Figure 1 displays a flowchart representing the data extraction and inclusion process. Five hundred ninety-nine members' charts were reviewed, of which data from 39 females who met the study eligibility criteria were used for analysis. Longitudinal CRF data for all 39 females were included in the analysis, and data for muscle strength were available for a subset of 19.

All fitness outcomes were analyzed by using linear models based on the findings of the Lowess curves and likelihood ratio tests indicating the best model. Baseline characteristics are presented in Table 1. The mean age at the time of enrollment was 65.0 years. Baseline VO_2_peak was 16.8 mL/kg/min, which is comparable to values reported from other studies of females with CVD^24^ and represents 64%–70% of values for age-matched females without CVD.^29^

**Table 1.** Baseline Characteristics of 39 Females Enrolled in the CR Program

Variable	n	Value
Age (years)	39	65.0 ± 8.7
Weight (kg)	33	74.0 ± 19.7
Height (cm)	33	160.5 ± 6.6
Reason for enrollment: *n* (%)	39	
Coronary bypass graft		12 (30.8)
Coronary angioplasty		5 (12.8)
Valve surgery		4 (10.3)
Myocardial infarction		8 (20.5)
Other		10 (25.6)
Time since event (years)	32	1.3 (1.7)
Enrollment duration (years)	39	6.2 ± 4.8
Resting values		
Heart rate (beats/min)	34	71 ± 11
Systolic blood pressure (mmHg)	39	135 ± 20
Diastolic blood pressure (mmHg)	39	77 ± 8
Peak values		
Heart rate (beats/min)	39	129 ± 25
% age-predicted maximum heart rate	39	80 ± 14
Systolic blood pressure (mmHg)	28	172 ± 21
Diastolic blood pressure (mmHg)	28	84 ± 24
VO_2_peak (mL/kg/min)	39	16.8 ± 5.1
1RM		
Chest press (kg)	19	39 ± 14
Seated back row (kg)	19	33 ± 11
Leg extension (kg)	19	32 ± 11

Values are means ± standard deviations for continuous variables, frequency (percentage) for categorical variables.

1RM, 1-repetition maximum; CR, cardiac rehabilitation.

Overall, the mean program enrollment duration was 6.2 years but on closer inspection, there were nine females (23%) with enrollment durations exceeding 10 years. As such, there were fewer observations toward the upper limits of enrollment time. Data were, thus, truncated at 5 years for CRF and 6 years for muscle strength for the analysis to preserve ≥70% available data and improve the generalizability of the mixed models.

Table 2 displays the computed changes in VO_2_peak and muscle strength over 5 and 6 years of enrollment, respectively. Table 3 shows the mixed-model analysis of the age-adjusted linear trajectory of VO_2_peak over 5 years of enrollment in CR. There were no interactions found between enrollment time and age at enrollment. According to Bayesian model statistics, the best fitting model was linear and included a random intercept [variance estimate (standard error) 14.8 (3.8), 95% confidence interval 8.89, 24.5] but no effect of a random slope, indicating inter-individual variability in baseline VO_2_peak but similar individual trajectories. Data based on the *β* coefficients from this model indicate that the VO_2_peak increased by 1.8%/year over enrollment time.

**Table 2.** Computed Changes in Cardiorespiratory Fitness (VO_2_peak) and Muscle Strength (1-Repetition Maximum) Since Enrollment

CRF	Baseline	1 year	5 years
VO_2_peak (mL/kg/min)	17.0 ± 0.7 (15.7, 18.4)	17.3 ± 0.7 (16.0, 18.6)	18.3 ± 0.8 (16.7, 19.9)

Muscle strength	Baseline	1 year	6 years
Chest press (kg)	39.0 ± 2.6 (33.9, 44.3)	39.3 ± 2.2 (35.1, 43.6)	40.6 ± 2.9 (34.9, 46.3)
Seated back row (kg)	33.1 ± 1.5 (30.1, 36.0)	33.8 ± 1.4 (31.0, 36.6)	37.3 ± 1.9 (33.5, 41.1)
Knee extension (kg)	32.6 ± 1.9 (28.9, 36.3)	33.1 ± 1.8 (29.5, 36.6)	35.4 ± 2.2 (31.1, 39.7)

Values are mean ± SE (95% CI).

CI, confidence interval; CRF, cardiorespiratory fitness; SE, standard error.

**Table 3.** Mixed-Model Analysis on Changes in VO_2_peak Levels in Females Older Than 5 Years of CR Enrollment After Controlling for Age (*n* = 39, 123 Observations)

Fixed and random-effects components from 0 to 5 years of enrollment			
Fixed-effects variables	β (SE)	95% CI	p
Time	0.26 (0.14)	−0.02, 0.54	0.072
Age at enrollment	−0.25 (0.08)	−0.40, −0.10	0.001^*^
Constant	33.1 (5.0)	23, 43	<0.0001^*^

Random-effects variance components	Estimate (SE)	95% CI	
Intercept	14.9 (3.9)	8.9, 24	
Residual	5.2 (0.8)	3.9, 7.1	

^*^ *p* < 0.05.

Table 4 shows the mixed-model analyses for age-adjusted trajectories for 1RM chest press, seated back row, and knee extension over 6 years of CR enrollment. Based on the Bayesian model statistics, linear models with random intercepts best captured the changes observed in seated back row and knee extension muscle strength, whereas the linear model with a random intercept, random slope, and an unstructured covariance structure best captured the trajectory of chest press.

**Table 4.** Mixed-Model Analysis on Changes in Muscle Strength for 1) Chest Press, 2) Seated Back Row, and 3) Knee Extension in Females Older Than 6 Years of CR Enrollment After Controlling for Age (*n* = 19, 66 Observations)

Fixed- and random-effects components from 0 to 6 years of enrollment			
1) Chest press			
Fixed-effects variables	β (SE)	95% CI	p
Time	0.25 (0.70)	−1.13, 1.62	0.725
Age at enrollment	−0.85 (0.20)	−1.24, −0.45	<0.0001^*^
Constant	94 (13)	68, 119	<0.0001^*^

Random-effects variance components	Estimate (SE)	95% CI	
Intercept	120.3 (44)	58.7, 247	
Slope (time)	6.9 (3.1)	2.9, 16.5	
Covariance (time, intercept)	−22.1 (10)	−42.0, −2.1	
Residual	16.3 (4.2)	9.9, 26.9	

2) Seated back row			
Fixed-effects variables	β (SE)	95% CI	p
Time	0.71 (0.32)	0.08, 1.33	0.026^*^
Age at enrollment	−0.55 (0.16)	−0.87, −0.24	0.001^*^
Constant	69 (10)	49, 89	<0.0001^*^

Random-effects variance components	Estimate (SE)	95% CI	
Intercept	29.7 (12)	13.3, 66.3	
Residual	20.0 (4.1)	13.4, 30.0	

3) Knee extension			
Fixed-effects variables	β (SE)	95% CI	p
Time	0.47 (0.31)	−0.13, 1.07	0.122
Age at enrollment	−0.64 (0.21)	−1.05, −0.24	0.002^*^
Constant	74 (13)	48, 100	<0.0001^*^

Random-effects variance components	Estimate (SE)	95% CI	
Intercept	53.8 (21)	24.8, 117	
Residual	18.2 (3.9)	12.0, 27.6	

^*^ *p* < 0.05.

There were no interactions between enrollment time and age. Muscle strength increased by 0.6 to 2.1%/year over 6 years, but it only reached statistical significance for the seated back row.

## Discussion

This study provides the initial evidence to suggest that females enrolled in a maintenance-phase CR program demonstrate trajectories of continuous improvements in both cardiovascular and musculoskeletal fitness over 5–6 years of enrollment.

The continued increase in CRF and muscle strength over time in females with CVD are in contrast not only to the non-linear changes previously reported among males enrolled in long-term CR^27^ but also to the expected declines in fitness associated with aging as reported in females without CVD.^29–31^ It is possible that the access to long-term CR may mitigate aging-related fitness declines in women. These findings underscore first, that sex-based considerations are needed in CR research and second, that sex-specific analysis can provide greater insight into the benefits of long-term CR for individuals living with CVD.

The possible mechanisms underlying the differential effects of long-term exercise on fitness between males and females are unknown, but sex- and gender-related differences in physiological and behavioral factors may provide some insight. Cardiac output and left ventricular mass,^32.33^ flow-mediated vasodilation,^34^ and muscle mass and strength^35^ decline to a lesser degree with aging in females compared with males, which may partially account for greater exercise-related benefits in fitness observed in females.

Sex hormones may also play a role, as the loss of testosterone in males after the age of 40 and the associated loss of skeletal muscle mass can contribute to accelerated declines in fitness.^36–39^ We note that in the current study, one-third of female participants were aged 40–59 years at program entry and thus may still have benefited from the cardio-protective effects of estrogen before menopause.^37,38^ Finally, the relative preservation of fat-free mass in aging females compared with males may also support analogous sex-specific preservation of fitness levels.^38^

Behavioral factors, such as program attendance and adherence, may also help to explain differences in fitness changes between men and women. It is known that women with CVD are less likely than men to be referred to, and participate in, CR programs^40,41^ but interestingly, among those who do enroll in CR, women are more likely to meet the recommended ≥150 minutes/week of moderate-intensity physical activity.^42^

Such behavioral factors associated with CR enrollment and adherence likely reflect gender-related differences that are beyond the scope of the current study, yet they underscore the importance of identifying strategies to overcome barriers to enrollment and participation in CR among women.

We acknowledge the limitations of this study. The sample size of 39 females limits the generalizability of the findings, and due to the retrospective nature of this analysis, we cannot infer causal links for the observed changes in fitness with CR enrollment in females. Insights could not be gained through program attendance, adherence or physical activity records, as this information was not available. We acknowledge that there may be a risk of sampling bias, as individuals who were less likely to demonstrate improvements in fitness may not have enrolled in CR or been able to continue with the program over the long term; however, information on loss to follow-up as a result of morbidity or mortality was not available.

The variance estimates, relative to the average slopes in the data, must be interpreted with caution as large between-participant variations in fitness trajectories were observed due to the small sample. Future studies with larger samples are needed to investigate these between-level changes in long-term CR programs.

However, this study also has several strengths. Despite the small sample size, our dataset provided a high number of observations over multiple time points, thereby permitting mixed-model analyses that adds to our understanding of long-term trajectories of fitness and that are superior to conventional pre–post measurement research designs.

The data in the current study further allowed for the identification of potential non-linear and random effects; this method of modeling provides a more complete picture of changes in fitness over time, thus improving the interpretability and generalizability of the findings. This was the first study to report on fitness trajectories over 5–6 years of CR in females, which was not possible in other studies of long-term CR that did not disaggregate data by sex.^24^

## Conclusions

Females are under-represented in CR research.^7^ Our results offer new, sex-specific evidence suggesting a trajectory of continued improved fitness in females with long-term enrollment in CR. Moreover, the improvements observed are in contrast with the expected declines in fitness that would be expected to occur with aging. These findings provide initial evidence to suggest the potential benefits of long-term exercise opportunities as strategies for secondary prevention in this high-risk population of females. Larger prospective cohort studies are warranted to confirm the observations, and to identify the potential mechanisms underlying these changes.

## Abbreviations Used

1RM: 1-repetition maximum
CI: confidence interval
CR: cardiac rehabilitation
CRF: cardiorespiratory fitness
CVD: cardiovascular disease
SE: standard error

## References

[1] Roth GA, Johnson C, Abajobir A, et al. Global, regional, and national burden of cardiovascular diseases for 10 causes, 1990 to 2015. J Am Coll Cardiol 2017;70:1–25. 28527533 10.1016/j.jacc.2017.04.052 PMC5491406

[2] Wei Y, George NI, Chang C, et al. Assessing sex differences in the risk of cardiovascular disease and mortality per increment in systolic blood pressure: A systematic review and meta-analysis of follow-up studies in the United States. PLoS One 2017;12:e0170218. 28122035 10.1371/journal.pone.0170218 PMC5266379

[3] Finks SW. Cardiovascular disease in women. Am Coll Clin Pharmacol 2010:179–199.

[4] Go AS, Mozaffarian D, Roger VL, et al. Heart disease and stroke statistics—2014 update: A report from the American Heart Association. Circulation 2014;129:e28–e292. 24352519 10.1161/01.cir.0000441139.02102.80 PMC5408159

[5] Roger VL, Go AS, Lloyd-Jones DM, et al. Heart disease and stroke statistics—2011 update: A report from the American Heart Association. Circulation 2011;123:e18–e209. 21160056 10.1161/CIR.0b013e3182009701 PMC4418670

[6] Johnston N, Schenck-Gustafsson K, Lagerqvist B. Are we using cardiovascular medications and coronary angiography appropriately in men and women with chest pain? Eur Heart J 2011;32:1331–1336. 21317147 10.1093/eurheartj/ehr009

[7] Anderson L, Oldridge N, Thompson DR, et al. Exercise-based cardiac rehabilitation for coronary heart disease: Cochrane systematic review and meta-analysis. J Am Coll Cardiol 2016;67:1–12. 26764059 10.1016/j.jacc.2015.10.044

[8] Kavanagh T, Mertens DJ, Hamm LF, et al. Prediction of long-term prognosis in 12 169 men referred for cardiac rehabilitation. Circulation 2002;106:666–671. 12163425 10.1161/01.cir.0000024413.15949.ed

[9] Kavanagh T, Mertens DJ, Hamm LF, et al. Peak oxygen intake and cardiac mortality in women referred for cardiac rehabilitation. J Am Coll Cardiol 2003;42:2139–2143. 14680741 10.1016/j.jacc.2003.07.028

[10] Nishitani M, Shimada K, Masaki M, et al. Effect of cardiac rehabilitation on muscle mass, muscle strength, and exercise tolerance in diabetic patients after coronary artery bypass grafting. J Cardiol 2013;61:216–221. 23332345 10.1016/j.jjcc.2012.11.004

[11] Sumide T, Shimada K, Ohmura H, et al. Relationship between exercise tolerance and muscle strength following cardiac rehabilitation: Comparison of patients after cardiac surgery and patients with myocardial infarction. J Cardiol 2009;54:273–281. 19782265 10.1016/j.jjcc.2009.05.016

[12] Sandercock G, Hurtado V, Cardoso F. Changes in cardiorespiratory fitness in cardiac rehabilitation patients: A meta-analysis. Int J Cardiol 2013;167: 894–902. 22206636 10.1016/j.ijcard.2011.11.068

[13] Santos KM, Neto ML, Carvalho VO, et al. Evaluation of peripheral muscle strength of patients undergoing elective cardiac surgery: A longitudinal study. Rev Bras Cir Cardiovasc 2014;29:355–359. 25372909 10.5935/1678-9741.20140043 PMC4412325

[14] Yamamoto S, Hotta K, Ota E, et al. Effects of resistance training on muscle strength, exercise capacity, and mobility in middle-aged and elderly patients with coronary artery disease: A meta-analysis. J Cardiol 2016;68:125–134. 26690738 10.1016/j.jjcc.2015.09.005

[15] Yang Y, He X, Gua H, et al. Efficacy of muscle strength training on motor function in patients with coronary artery disease: A meta-analysis. Int J Clin Exp Med 2015;8:17536–17550. 26770345 PMC4694245

[16] Mikhail G. Coronary heart disease in women is underdiagnosed, undertreated, and under-researched. BMJ 2005;331:467–468. 16141136 10.1136/bmj.331.7515.467 PMC1199011

[17] Feola M, Garnero S, Daniele B, et al. Gender differences in the efficacy of cardiovascular rehabilitation in patients after cardiac surgery procedures. J Geriatr Cardiol 2015;12:575–579. 26512250 10.11909/j.issn.1671-5411.2015.05.015 PMC4605954

[18] Gupta R, Sanderson B, Bittner V. Outcomes at one-year follow-up of women and men with coronary artery disease discharged from cardiac rehabilitation. J Cardiopulm Rehabil Prev 2007;27:11–18. 17474639 10.1097/01.hcr.0000265015.44210.bf

[19] Colbert JD, Martin BJ, Haykowsky MJ, et al. Cardiac rehabilitation referral, attendance and mortality in women. Eur J Prev Cardiol 2015;22:979–986. 25278001 10.1177/2047487314545279

[20] Balady G, Jette D, Scheer J, et al. Changes in exercise capacity following cardiac rehabilitation in patients stratified according to age and gender: Results of the Massachusetts Association of Cardiovascular and Pulmonary Rehabilitation Multicenter Database. J Cardiopulm Rehabil 1996;16:38–46. 8907441 10.1097/00008483-199601000-00005

[21] Wise FM, Patrick JM. Cardiac rehabilitation in women with chronic heart failure: Mood, fitness, and exercise safety. J Cardiopulm Rehabil Prev 2012;32:78–84. 22354014 10.1097/HCR.0b013e3182460c4b

[22] Cannistra LB, Balady GJ, O'Malley CJ, et al. Comparison of the clinical profile and outcome of women and men in cardiac rehabilitation. Am J Cardiol 1992;69:1274–1279. 1585859 10.1016/0002-9149(92)91220-x

[23] Norris CM, Yip CY, Nerenberg KA, et al. State of the science in women's cardiovascular disease: A Canadian perspective on the influence of sex and gender. J Am Heart Assoc 2020;9:e015634. 32063119 10.1161/JAHA.119.015634 PMC7070224

[24] Ghishi GLM, Chaves GSDS, Bennett A, et al. The effects of cardiac rehabilitation on mortality and morbidity in women: A meta-analysis attempt. J Cardiopulm Rehabil Prev 2019;39:39–42. 30252785 10.1097/HCR.0000000000000351

[25] Gayda M, Juneau M, Levesque S, et al. Effects of long-term and ongoing cardiac rehabilitation in elderly patients with coronary heart disease. Am J Geriatr Cardiol 2006;15:345–351. 17086026 10.1111/j.1076-7460.2006.05245.x

[26] Belardinelli R, Georgiou D, Cianci G, et al. 10-year exercise training in chronic heart failure a randomized controlled trial. J Am Coll Cardiol 2012;60:1521–1528. 22999730 10.1016/j.jacc.2012.06.036

[27] Pryzbek M, MacDonald M, Stratford P, et al. Long-term enrollment in cardiac rehabilitation benefits cardiorespiratory fitness and skeletal muscle strength in men with cardiovascular disease. Can J Cardiol 2019;35:1359–1365. 31495685 10.1016/j.cjca.2019.05.018

[28] Stone J, Arthur H, Suskin N, et al. Canadian Guidelines for Cardiac Rehabilitation and Cardiovascular Disease Prevention: Translating knowledge into action, 3rd ed. Winnipeg, Manitoba: Canadian Association of Cardiac Rehabilitation, 2009.

[29] Fleg JL, Morrell CH, Bos AG, et al. Accelerated longitudinal decline of aerobic capacity in healthy older adults. Circulation 2005;112:674–682. 16043637 10.1161/CIRCULATIONAHA.105.545459

[30] Keller K, Engelhardt M. Strength and muscle mass loss with aging process. Age and strength loss. Muscles Ligaments Tendons J 2014;3:346–350. 24596700 PMC3940510

[31] von Haehling S, Morley JE, Anker SD. An overview of sarcopenia: Facts and numbers on prevalence and clinical impact. J Cachexia Sarcopenia Muscle 2013;1:129–133. 10.1007/s13539-010-0014-2 PMC3060646 21475695

[32] Weiss EP, Spina RJ, Holloszy JO, et al. Gender differences in the decline in aerobic capacity and its physiological determinants during the later decades of life. J Appl Physiol 2006;101:938–944. 16497840 10.1152/japplphysiol.01398.2005

[33] Goldspink DF, George KP, Chantler PD, et al. A study of presbycardia, with gender differences favoring ageing women. Int J Cardiol 2009;137:236–245. 18718688 10.1016/j.ijcard.2008.06.086

[34] Black MA, Cable NT, Thijssen DH, et al. Impact of age, sex, and exercise on brachial artery flow-mediated dilatation. Am J Physiol Heart Circ Physiol 2009;297:H1109–H1116. 19633208 10.1152/ajpheart.00226.2009 PMC2755978

[35] Goodpaster BH, Won Park S, Harris TB, et al. The loss of skeletal muscle strength, mass, and quality in older adults: The Health, Aging and Body Composition Study. J Gerontol 2005;61:1059–1064. 10.1093/gerona/61.10.1059 17077199

[36] Brown M. Skeletal muscle and bone: Effect of sex steroids and aging. Adv Physiol Educ 2008;32:120–126. 18539850 10.1152/advan.90111.2008

[37] Parker BA, Kalasky MJ, Proctor D. Evidence for sex differences in cardiovascular aging and adaptive responses to physical activity. Eur J Appl Physiol 2010;110:235–246. 20480371 10.1007/s00421-010-1506-7 PMC2929283

[38] Hughes VA, Frontera WR, Roubenoff R, et al. Longitudinal changes in body composition in older men and women: Role of body weight and physical activity. Am J Clin Nutr 2002;76:473–481. 12145025 10.1093/ajcn/76.2.473

[39] Visser M, Kritchevsky SB, Goodpaster BH, et al. Leg muscle mass and composition in relation to lower extremity performance in men and women aged 70 to 79: The health, aging and body composition study. J Am Geriatr Soc 2002;50:897–904. 12028178 10.1046/j.1532-5415.2002.50217.x

[40] Samayoa L, Grace SL, Gravely S, et al. Sex differences in cardiac rehabilitation enrollment: A meta-analysis. Can J Cardiol 2014;30:793–800. 24726052 10.1016/j.cjca.2013.11.007

[41] Supervia M, Medina-Inojosa JR, Yeung C, et al. Cardiac rehabilitation for women: A systematic review of barriers and solutions. Mayo Clinic Proc 2017;92:565–577. 10.1016/j.mayocp.2017.01.002 PMC5597478 28365100

